# Supplementary material for: Exercise training remodels inter-organ endocrine networks
Source: bioRxiv. 2025 May 4:2025.04.29.651323. Preprint. [Version 1] doi: 10.1101/2025.04.29.651323 (PMC12248110; doi:10.1101/2025.04.29.651323)
Supplement: 1 [file NIHPP2025.04.29.651323V1-supplement-1.pdf]

Supplementary Fig 1A

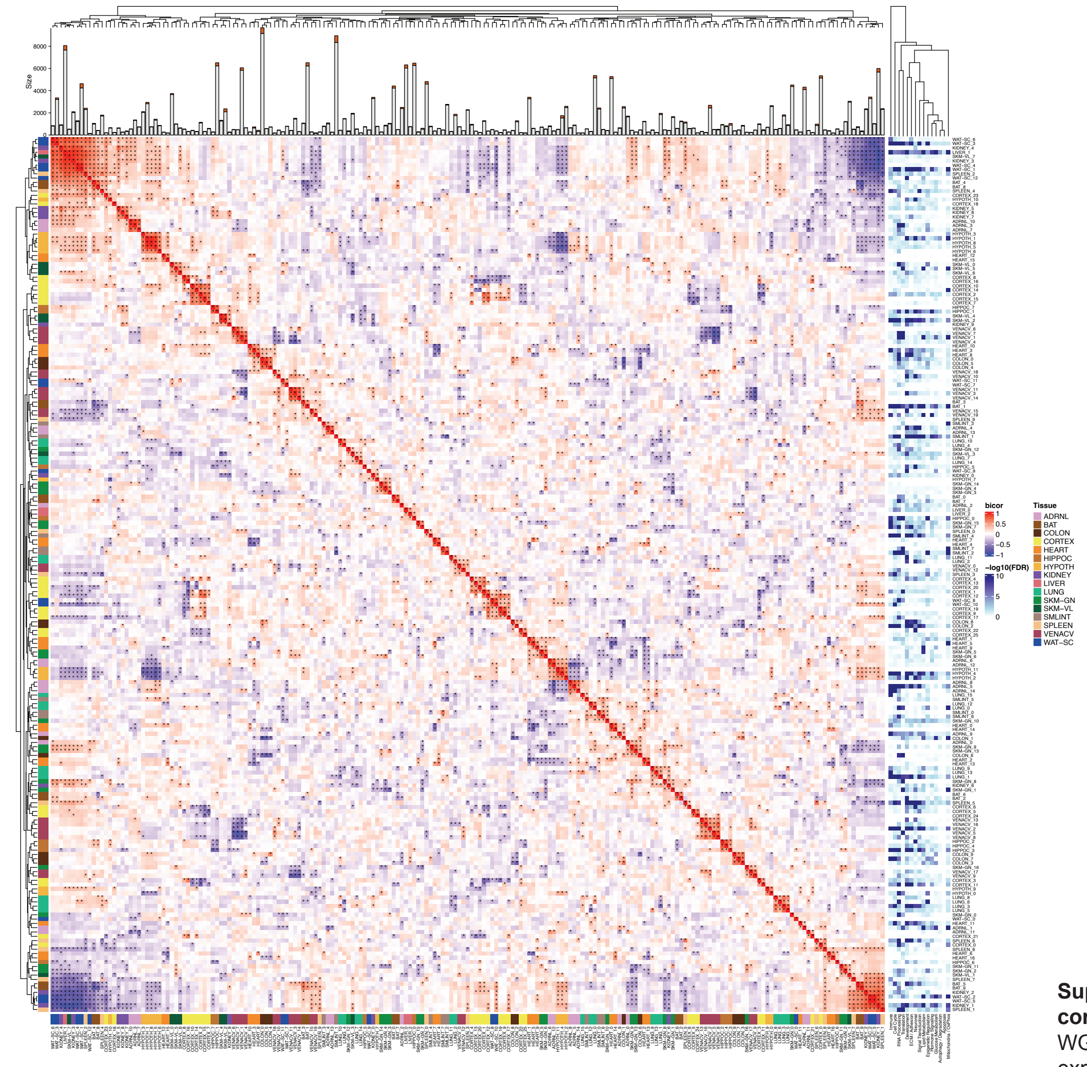

Supplementary Fig 1B

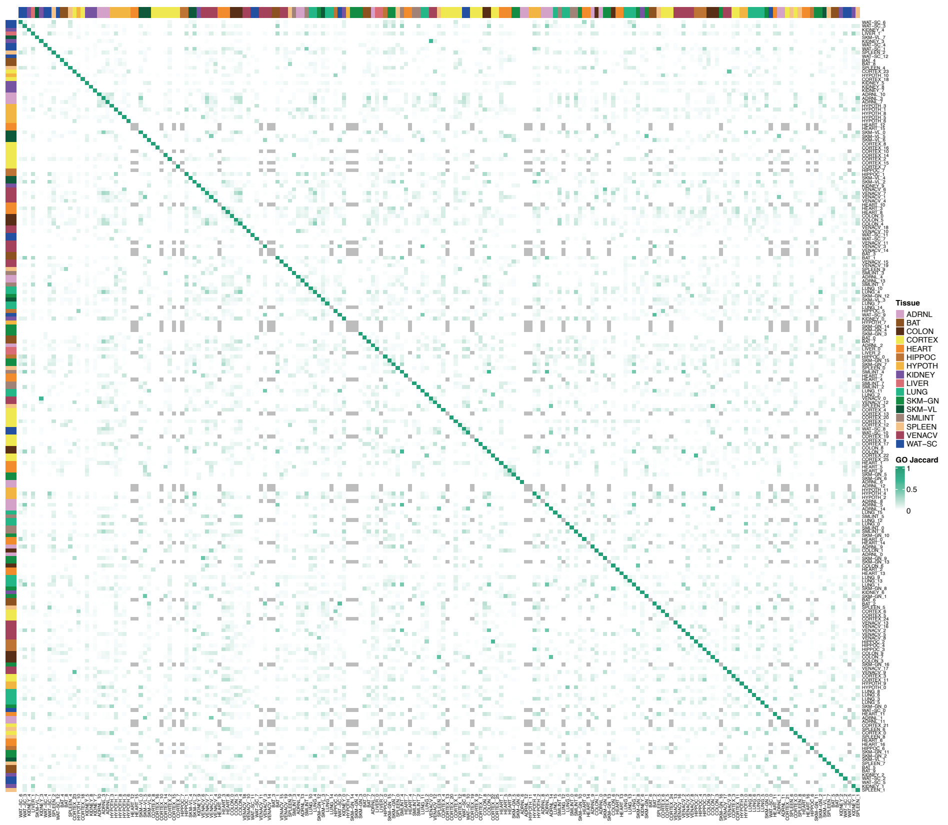

Supplementary Figure 1. Module-to-module correlations across tissue transcriptomes
WGCNA was performed on concatenated gene expression matrices from all samples (n=50 rats) for each tissue, yielding a total of 203 co-expression modules across 16 tissues.

A) ComplexHeatmap showing pairwise correlations between module eigengenes. *Adjusted p<0.05. Bar plots above each column represent module size (i.e., number of genes per module), with the orange portion indicating the number of secretory genes. Over-representation analysis (ORA) results are shown on the right. To reduce redundancy and highlight key biological themes, significantly enriched GO terms were manually curated into categories, including: Translation, Mitochondria/Oxphos, Immune, Neuronal, Cell Cycle, RNA Processing, Lipid Metabolism, Glucose Metabolism, Development, Apoptosis, Autophagy/Degradation, Epigenetic Regulation, Signal Transduction, ECM/Adhesion, and Hormone Signaling (see Methods for classification pipeline).

B) Heatmap showing the Jaccard index (range: 0–1) for overlap of significantly enriched GO terms (FDR<0.05) between modules. Higher Jaccard values indicate greater overlap in functional enrichment. Row and column order mirrors that of panel A. Gray boxes indicate modules with no significantly enriched terms.

Supplementary Fig 2A

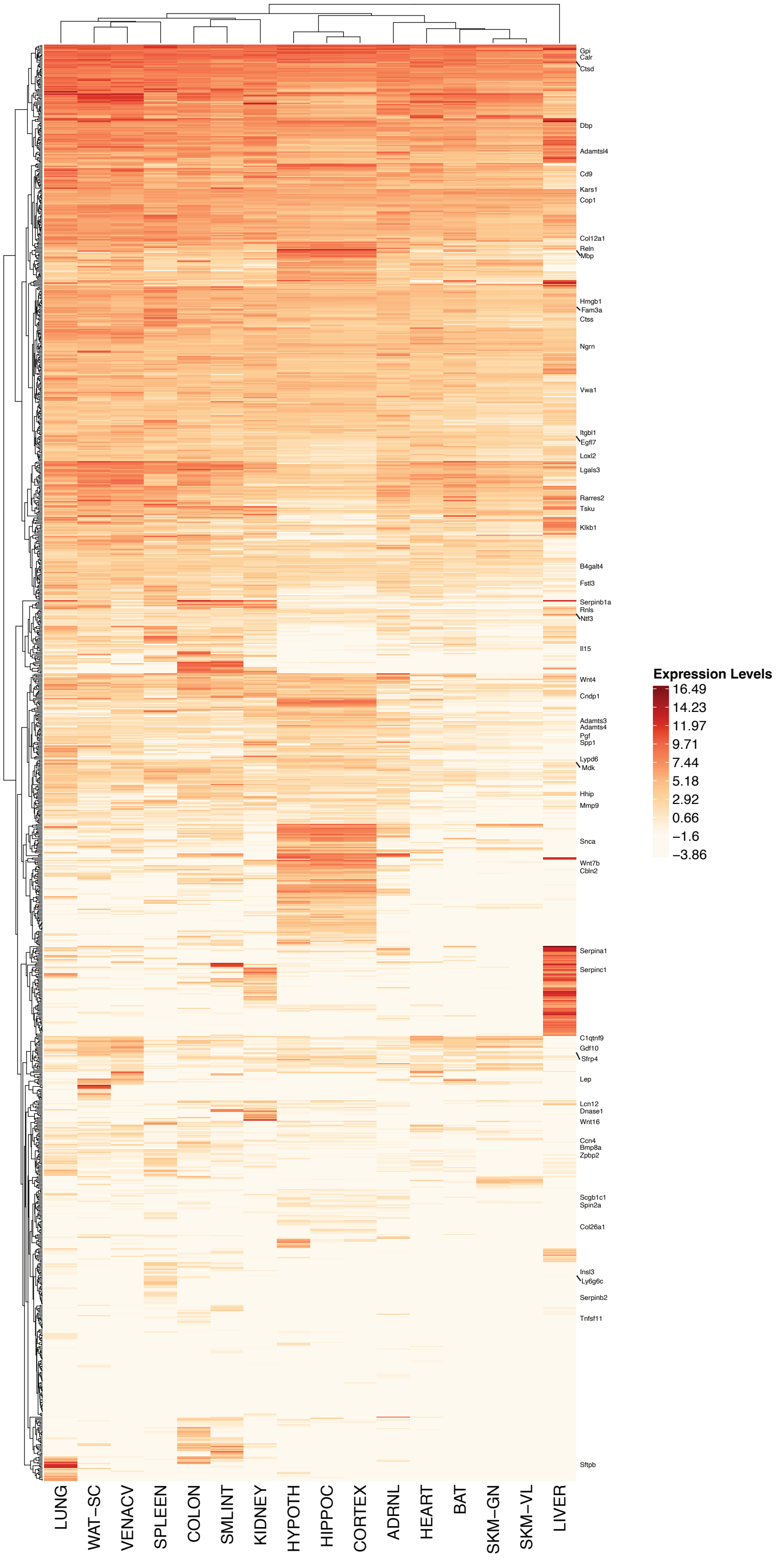

Supplementary Fig 2B

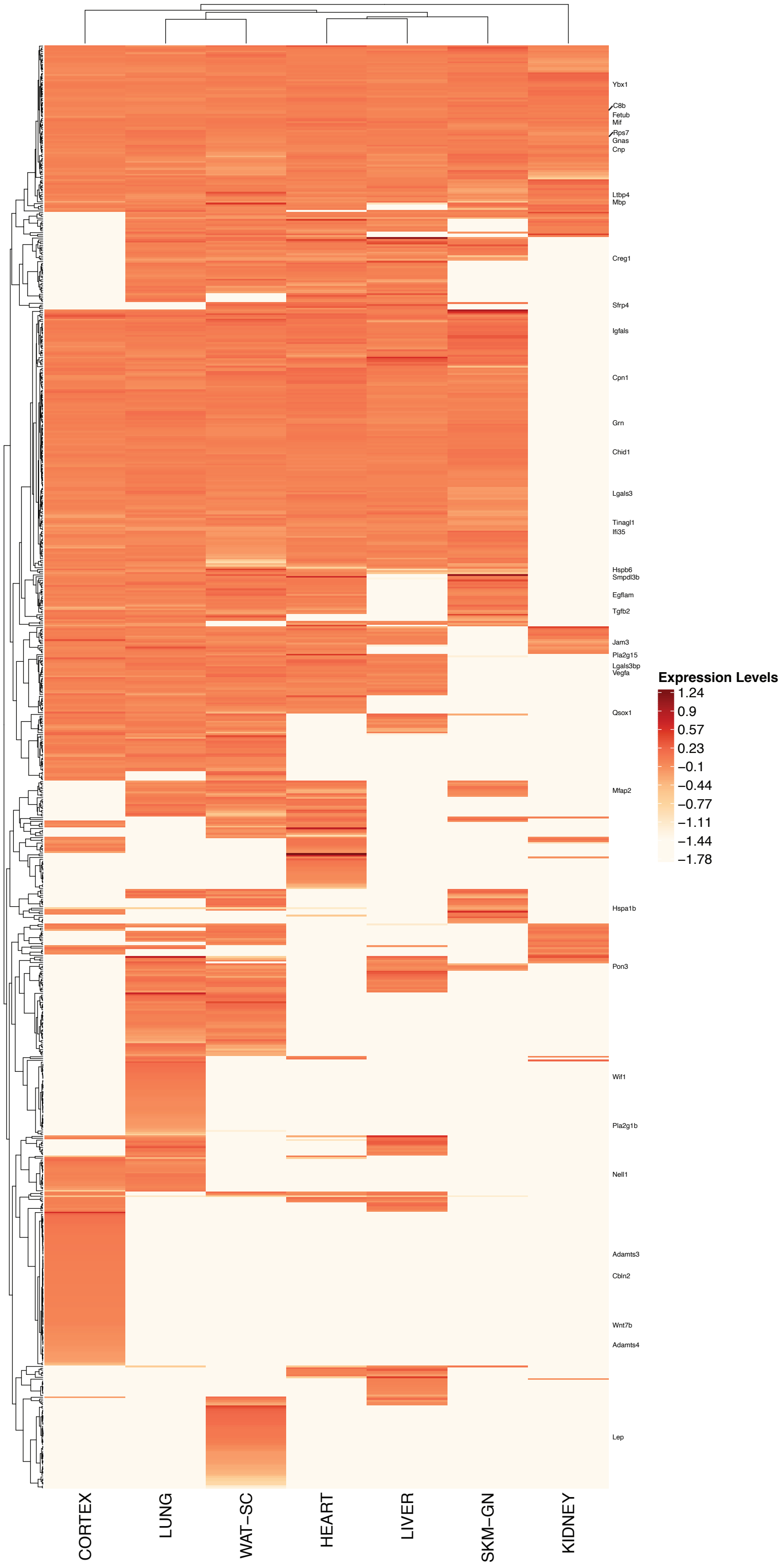

Supplementary Figure 2. Secretory transcript expression and protein abundance across tissues.
A) Heatmap of raw expression (log2 CPM) of 1117 secretory transcripts across 16 tissues. Undetected transcripts were imputed as the lowest expression level. B) Heatmap of raw abundance (log2 normalized abundance) of 797 secretory proteins across 7 tissues. Undetected proteins were imputed as the lowest abundance.

Supplementary Fig 3A

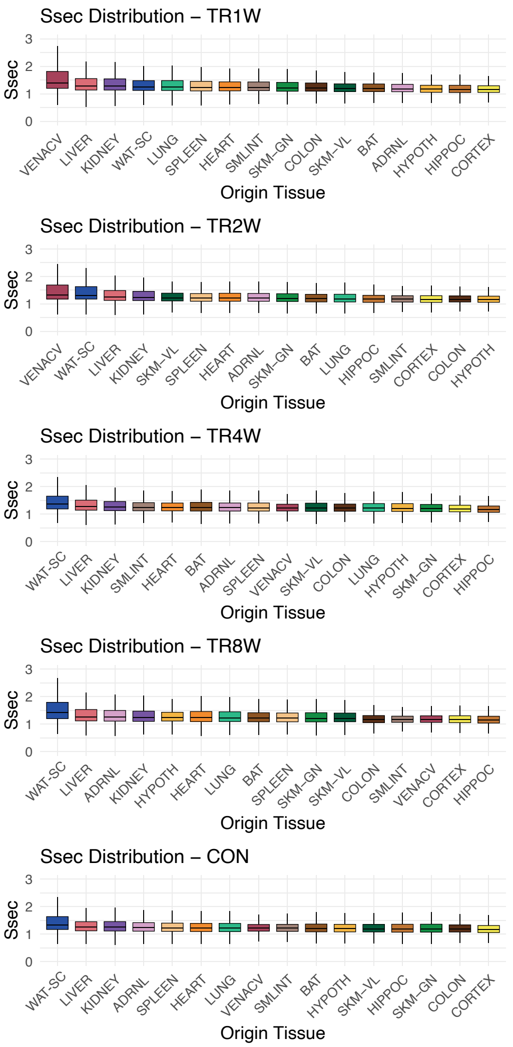

Supplementary Fig 3B

Transcriptomics

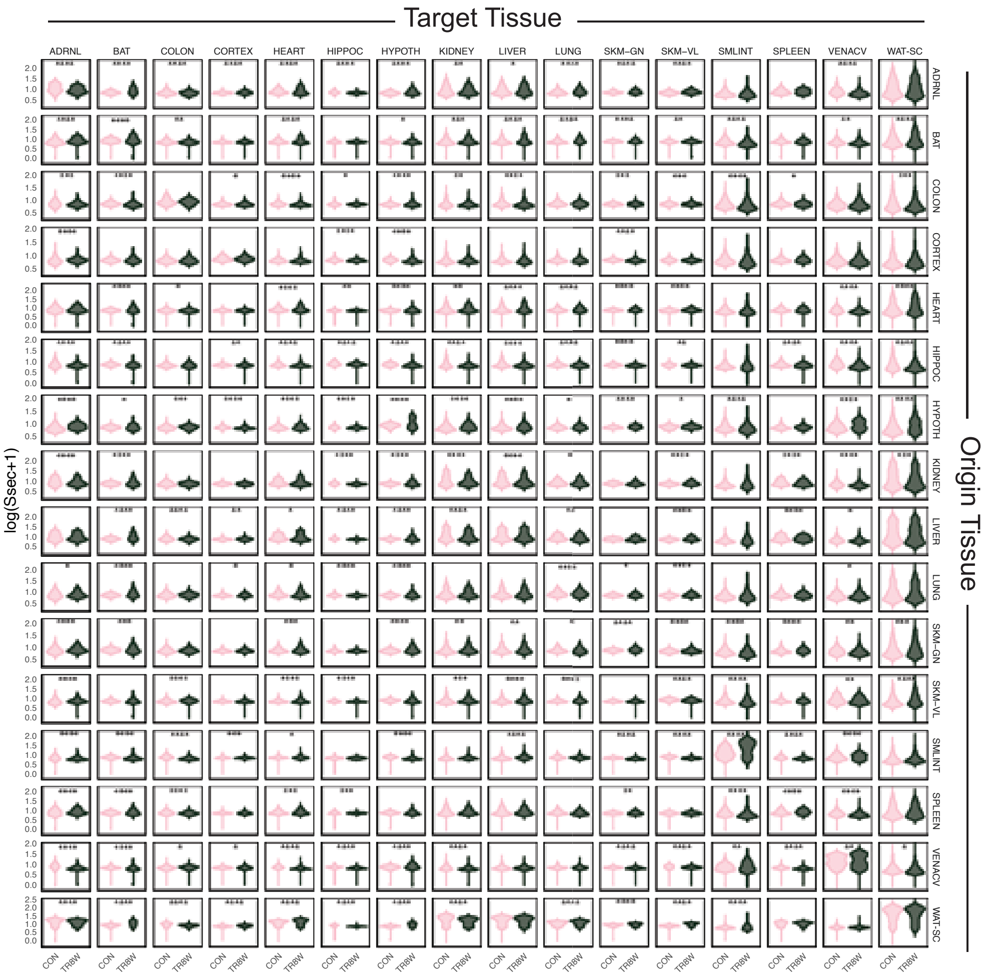

Supplementary Fig 3C

Proteomics

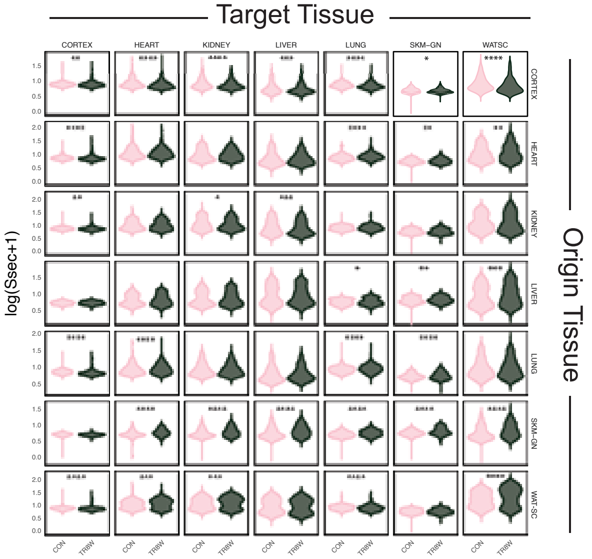

Supplementary Figure 3. Ssec distribution over training and comparision of Ssec in CON vs. TR8W
A) Boxplots of tissue-specific Ssec across groups. B) Comparision of Ssec in CON vs. TR8W in all possible origin-target gene-to-gene correlation pairs. Paired T-test was performed on log(x+1) transformed Ssec. C) Comparision of Ssec in CON vs. TR8W in all possible origin-target protein-to-protein correlation pairs. Paired T-test was performed on log(x+1) transformed Ssec. *adjusted p<0.05, **adjusted p<0.01, ***adjusted p<0.001, ****adjusted p<0.0001. CON, control; TR1W, 1-week training, TR2W, 2-week training; TR4W, 4-week training; TR8W, 8-week training.

Supplementary Fig 4A

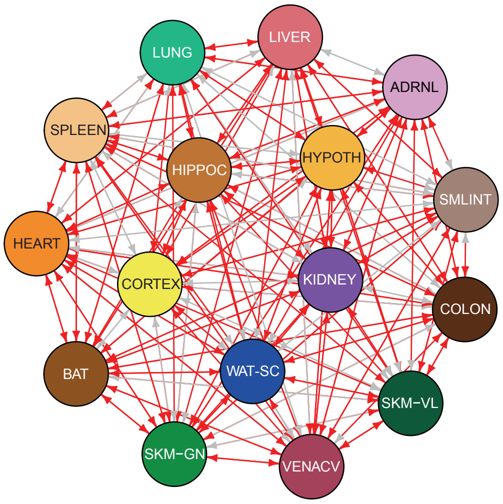

Supplementary Fig 4B

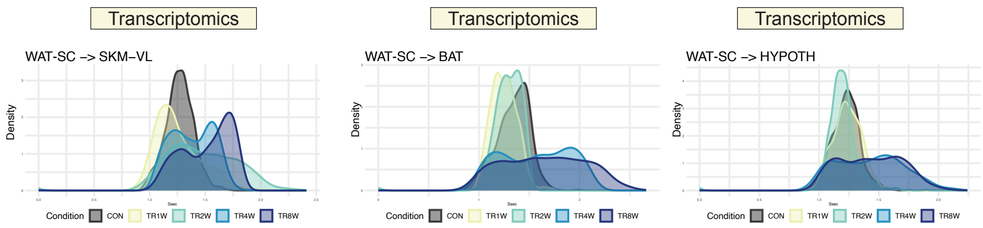

Supplementary Fig 4C

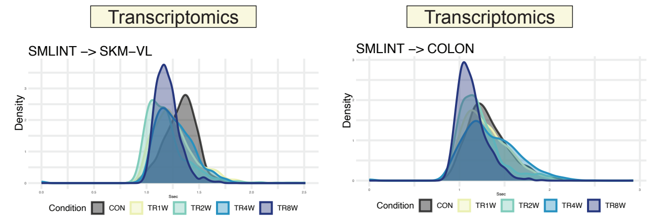

Supplementary Fig 4D

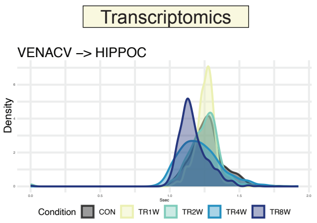

Supplementary Fig 4E

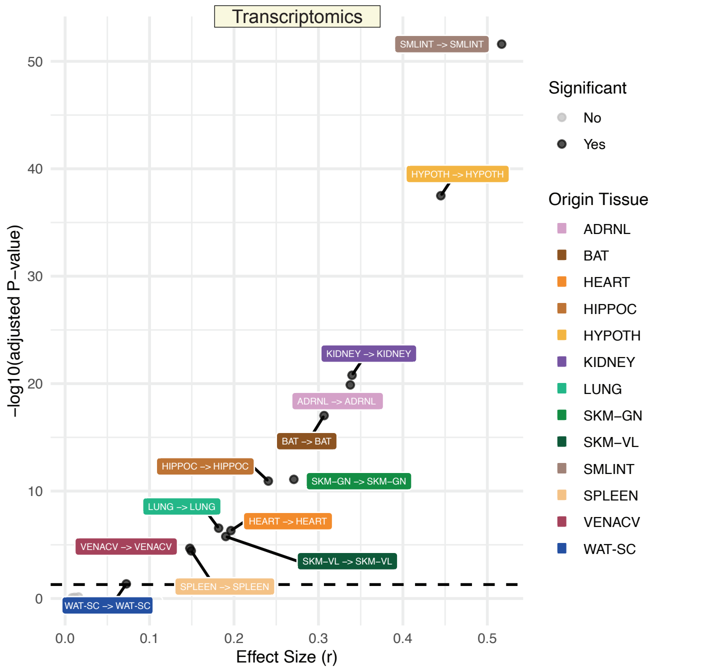

Supplementary Fig 4F

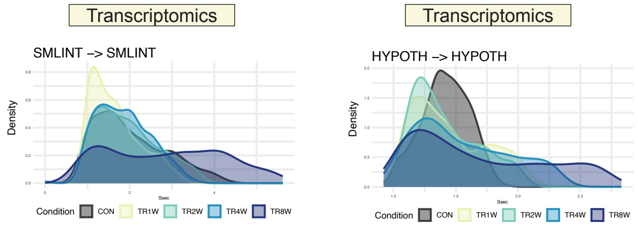

Supplementary Fig 4H

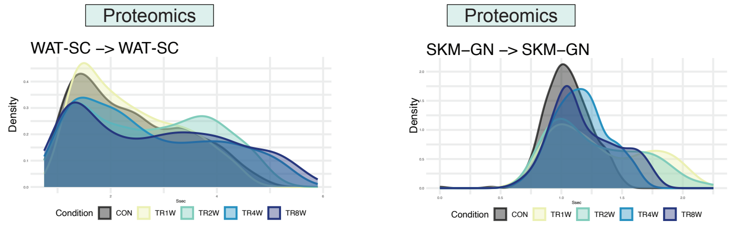

Supplementary Fig 4G

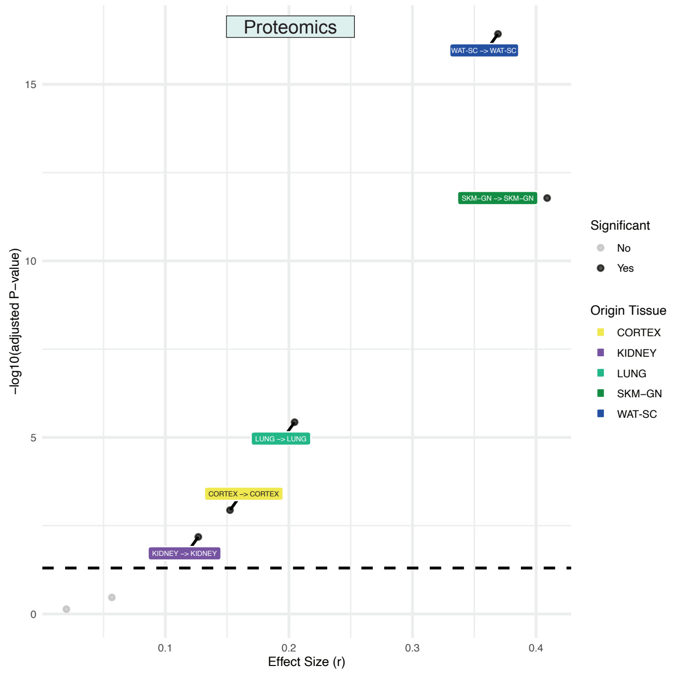

Supplementary Figure 4. Comparision of Ssec rankings in CON vs. TR8W

A) Network plot showing significantly different Ssec ranking in origin-target pairs in CON vs. TR8W. Red arrows represent significant difference (adjusted p<0.05). B) Density plots of WAT-SC-originating (target: SKM-VL, BAT, and HYPOTH) Ssec across groups. C) Density plots of SMLINT-originating (target: SKM-VL and COLON) Ssec across groups. D) Density plots of a VENACV-to-HIPPOC Ssec across groups. E) Volcano plot of wilcoxon signed rank test results comparing gene-to-gene Ssec ranking of matched origin-target tissue pairs in CON vs. TR8W. F) Density plots of SMLINT-to-SMLINT and HYPOTH-to-HYPOTH Ssec across groups (gene-to-gene). G) Volcano plot of wilcoxon signed rank test results comparing protein-to-protein Ssec ranking of matched origin-target tissue pairs in CON vs. TR8W. H) Density plots of WAT-SC-to-WAT-SC and SKM-GN-to-SKM-GN Ssec across groups (protein-to-protein).

Supplementary Fig 5A

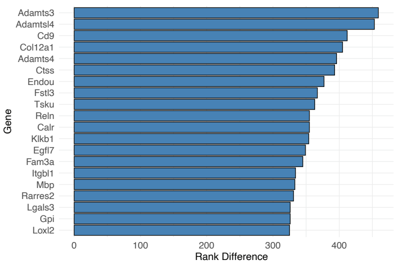

Supplementary Fig 5B

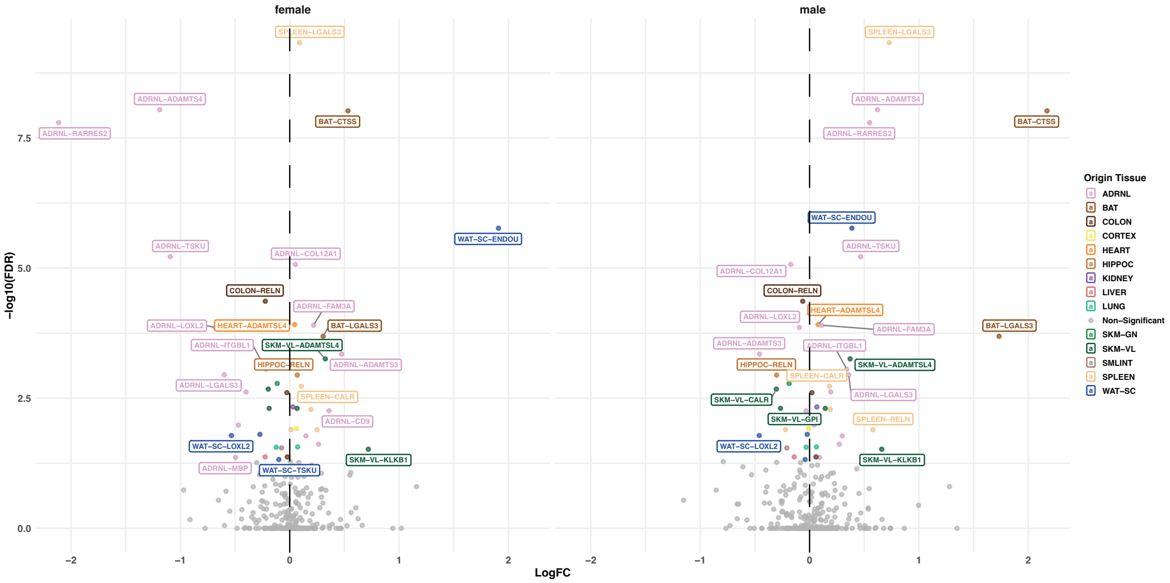

Supplementary Fig 5C

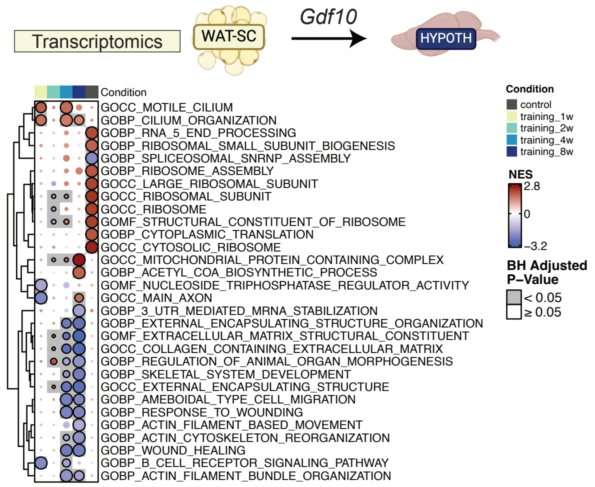

Supplementary Fig 5D

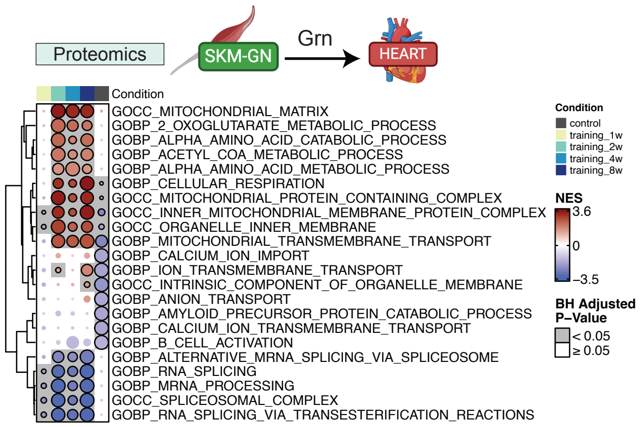

Supplementary Figure 5. Top different global and tissue-specific secretory transcript
A) Top 20 global secretory transcripts with largest Ssec ranking difference between CON and TR8W. The ranking differences are shown in bar plots. B) Volcano plots showing differentially regulated transcripts (FDR<0.05) by 8-week training among top 20 global transcripts with most Ssec rank difference between TR8W and CON. Given that the differential analysis was conducted in each sex by MoTrPAC, volcano plots were created within each sex. Fgsea results of C) hypothalamus transcripts correlating with scWAT-derived Gdf10 and D) heart proteins correlating with gastrocnemius-dervied Grn.

Supplementary Fig 6A

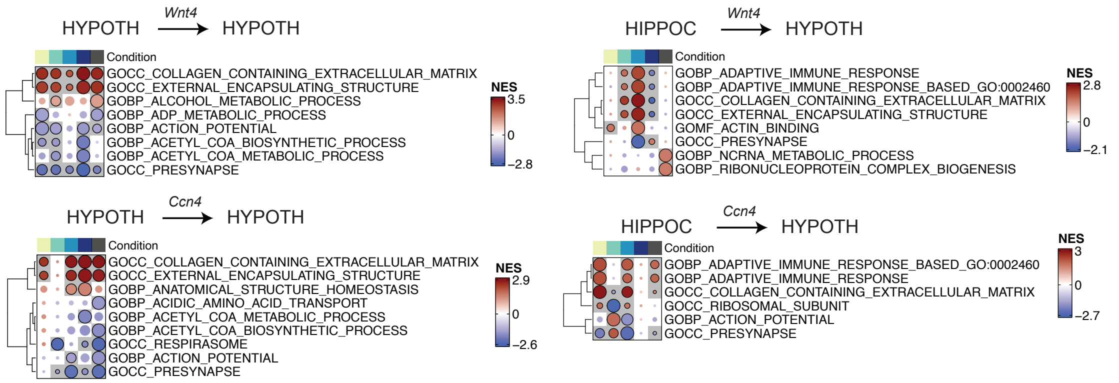

Supplementary Fig 6B

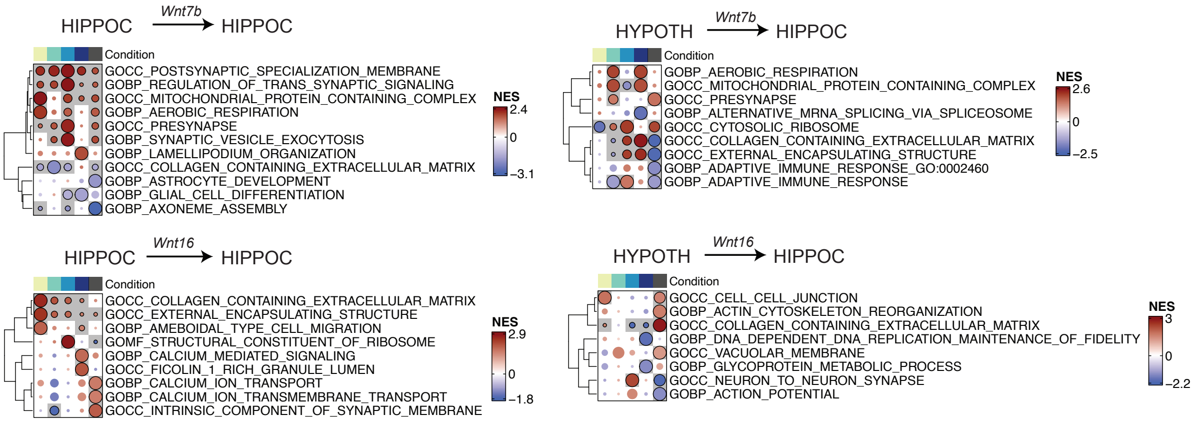

Supplementary Figure 6. GD-CAT analysis of autocrine and paracrine signaling by secretory Wnt factors in the hypothalamus and hippocampus.
A) Fgsea results of hypothalamus transcripts correlating with 1) hypothalamus Wnt4, 2) hippocampus Wnt4, 3) hypothalamus Ccn4, and 4) hippocampus Ccn4.
B) Fgsea results of hippocampus transcripts correlating with 1) hippocampus Wnt7b, 2) hypothalamus Wnt7b, 3) hippocampus Wnt16, and 4) hypothalamus Wnt16.
